# The elephant brain in numbers

**DOI:** 10.3389/fnana.2014.00046

**Published:** 2014-06-12

**Authors:** Suzana Herculano-Houzel, Kamilla Avelino-de-Souza, Kleber Neves, Jairo Porfírio, Débora Messeder, Larissa Mattos Feijó, José Maldonado, Paul R. Manger

**Affiliations:** ^1^Instituto de Ciências Biomédicas, Universidade Federal do Rio de JaneiroRio de Janeiro, Brazil; ^2^Instituto Nacional de Neurociência TranslacionalSão Paulo, Brazil; ^3^MBF Bioscience, Inc.Rio de Janeiro, Brazil; ^4^School of Anatomical Sciences, Faculty of Health Sciences, University of the WitwatersrandJohannesburg, South Africa

**Keywords:** elephant, cerebral cortex, numbers of neurons, neuronal density, cerebellum, glia/neuron ratio, brain size

## Abstract

What explains the superior cognitive abilities of the human brain compared to other, larger brains? Here we investigate the possibility that the human brain has a larger number of neurons than even larger brains by determining the cellular composition of the brain of the African elephant. We find that the African elephant brain, which is about three times larger than the human brain, contains 257 billion (10^9^) neurons, three times more than the average human brain; however, 97.5% of the neurons in the elephant brain (251 billion) are found in the cerebellum. This makes the elephant an outlier in regard to the number of cerebellar neurons compared to other mammals, which might be related to sensorimotor specializations. In contrast, the elephant cerebral cortex, which has twice the mass of the human cerebral cortex, holds only 5.6 billion neurons, about one third of the number of neurons found in the human cerebral cortex. This finding supports the hypothesis that the larger absolute number of neurons in the human cerebral cortex (but not in the whole brain) is correlated with the superior cognitive abilities of humans compared to elephants and other large-brained mammals.

## Introduction

What explains the superior cognitive abilities of the human brain, with richly complex and flexible behaviors, compared to other brains (Premack, 2007), some even larger than ours (Roth and Dicke, 2005)? Neuroanatomical correlates have been sought in total brain mass, cerebral cortical mass and cortical folding index, but these values are actually smaller in the human brain than in several other species (reviewed in Herculano-Houzel, 2011a). The elephant brain, in particular, at 4.5–5 kg, is about 3–4 times larger than the human brain (Manger et al., 2009). Another possibility was the relative mass of the cerebral cortex measured as a percentage of brain mass—but although this value is indeed largest in the human brain, it is only marginally so (Herculano-Houzel, 2011a). Encephalization quotient (EQ), the ratio between actual brain mass and that expected from the species' body mass, given the mathematical relationship between brain mass and body mass, has been considered the best proxy for cognitive abilities since its definition by Jerison (1973), because this is the parameter that singles out humans as the species with by far the largest value, around 7–8 (Jerison, 1973; Marino, 1998). The African elephant, by comparison, has an EQ of 1.3 (Roth and Dicke, 2005), and cetaceans have EQs of around 3 (Marino, 1998). However, the small EQ of the African elephant, similar to that of a walrus, camel, or squirrel (Roth and Dicke, 2005), does not seem to reflect observed behavioral abilities (Plotnik et al., 2006, 2011; Hart et al., 2008)—just like the higher EQ of capuchin monkeys compared to great apes is at odds with the greater behavioral flexibility and cognitive abilities of the latter (Deaner et al., 2007).

In contrast, and building on the assumption that neurons are the basic information-processing units of the brain, we have proposed that it is not the degree of encephalization, but rather the combined absolute number of neurons in the cerebral cortex and cerebellum, regardless of brain or body size, that correlates best with cognitive abilities (Herculano-Houzel, 2009, 2011a). Compared to other primates, and to smaller-brained species such as rodents, the human brain has indeed a much larger number of neurons, both in the cerebral cortex and in the cerebellum (Herculano-Houzel, 2012); however, testing the hypothesis that absolute numbers of neurons correlate with cognitive abilities across species, including humans, requires determining the cellular composition of brains larger than the human brain.

Here we determine the cellular composition of the brain of one adult male African elephant using the isotropic fractionator (Herculano-Houzel and Lent, 2005), a quantitative method that has been shown to yield similar results to stereology, but in much less time (Bahney and von Bartheld, 2014), and that does not require that analysis be limited to isotropic structures.

## Results and discussion

We find that the brain of the African elephant, weighing 4618.6 g (without the olfactory bulb), about 3 times heavier than the human brain, holds a total of 257.0 billion neurons, also 3 times more than the average of 86 billion neurons found in the human brain (Azevedo et al., 2009). Despite this, the cerebral cortex of the elephant brain, which weighs 2848.0 g (gray and white matter combined), more than two times the mass of the human cerebral cortex, is composed of only 5.6 billion neurons, which amounts to only about one third of the average 16.3 billion neurons found in the human cerebral cortex (Azevedo et al., 2009). Within the cerebral cortex, the elephant hippocampus weighs 24.42 g and has a slightly larger volume than the human hippocampus (Patzke et al., 2013), but holds only 36.63 million neurons bilaterally, compared to approximately 250 million neurons in the ensemble of the human hippocampus plus amygdala (Andrade-Moraes et al., 2013). The rest of the brain structures, excluding the cerebral cortex and cerebellum, holds as many neurons in the elephant brain as in the human brain: 0.7 billion neurons, despite being 4 times larger in the elephant brain (564.7 g compared to 117.7 g in the human brain). The vast majority of the neurons in the African elephant brain, 97.5%, are located in the cerebellum, which holds 250.7 billion neurons (Table 1).

**Table 1:** **Cellular composition of the African elephant brain**.

**Structure**	**Mass, g**	**Neurons**	**Other cells**	**N/mg**	**O/mg**	**O/N**
Whole brain	4,618.62	257.04 B	216.06 B	56,071	47,130	0.841
Cerebral cortex, GM + WM^*^	2,847.95	5.59 B	150.15 B	1964	52,721	26.844
Cerebral cortex, GM^**^	1,431.50	5.53 B	55.70 B	3.661	38,910	10.628
Cerebral cortex, WM	1,382.09	–	91.99 B	–	66,561	–
Cerebellum	1,171.63	250.71 B	38.51 B	213,983	32,866	0.154
Rest of brain	564.67	0.74 B	27.40 B	1314	48,531	36,948
Hippocampus	24.42	36.63 M	1.73 B	1500	70,714	47.154
Amygdala	9.94	28.46 M	0.73 B	2864	73,107	25.530
Striatum	242.38	330.75 M	13.65 B	1365	56,326	41.277
Diencephalon	91.18	97.20 M	5.27 B	1066	57,770	54.195
Mesencephalon	54.10	108.78 M	2.49 B	2011	45,976	22.867
Pons	102.00	123.53 M	3.16 B	1211	30,993	25.594
Medulla	74.99	81.44 M	2.83 B	1086	37,803	34.811
Olfactory bulb	41.88	908.37 M	2.86 B	21,687	68,230	3.146

All figures refer to 2 times the values obtained for one half-brain. N/mg, density of neurons per milligram of tissue. O/mg, density of other (non-neuronal) cells per milligram of tissue. B, billion cells (1 × 10^9^). M, million cells (1 × 10^6^). GM, gray matter. WM, white matter.

* , includes hippocampus and amygdala.

** *, excludes hippocampus and amygdala*.

**Figure 1:**
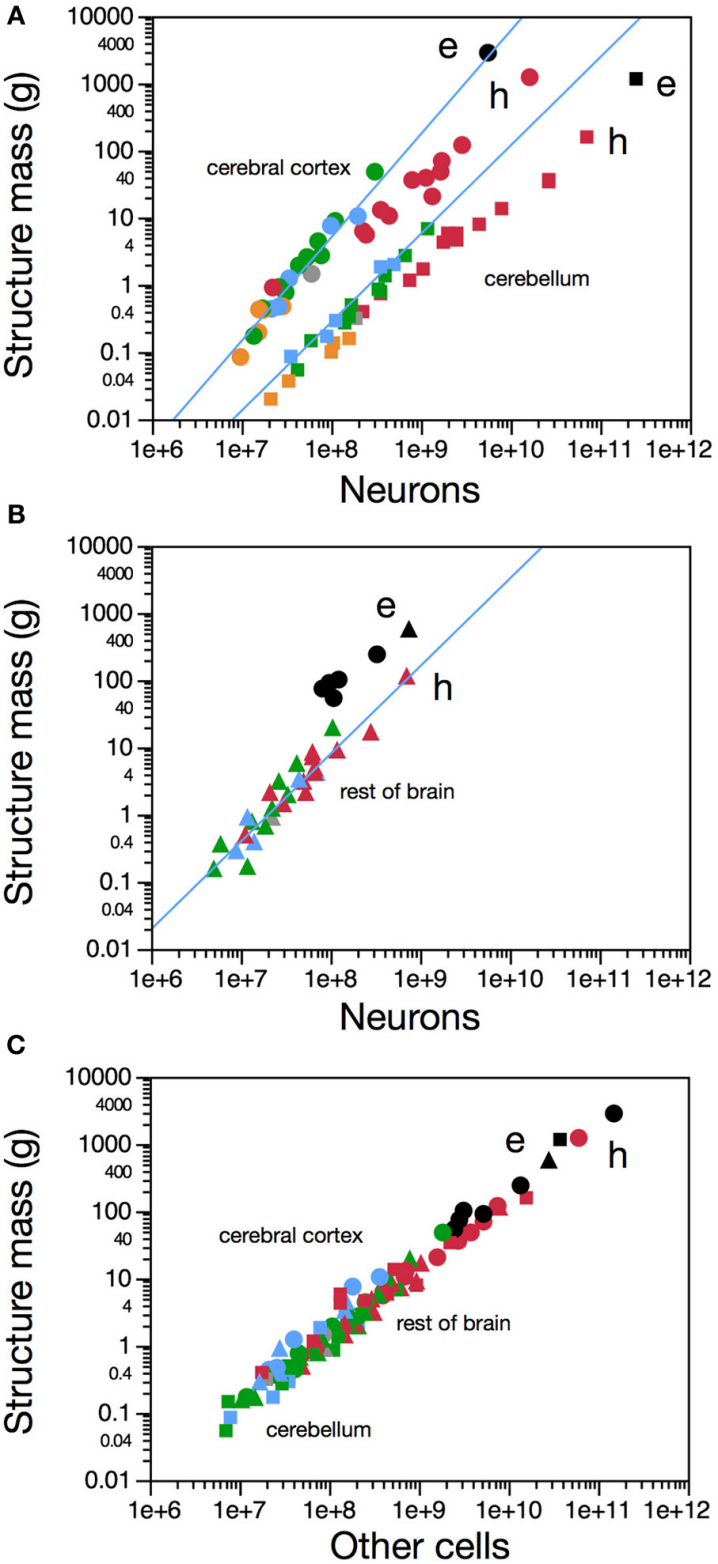
**The African elephant brain has as many neurons in the cerebral cortex as expected for an afrotherian of its cortical mass, but more neurons in the cerebellum than expected for its cerebellar mass, and fewer neurons in the remaining brain areas than expected for their mass**. In all figures, each data point represents values for both brain sides in one species. **(A)** Variation in mass of the cerebral cortex (gray and white matter combined; circles) and of the cerebellum (squares) as a function of the number of neurons in the respective structures. **(B)** Variation in mass of the remaining brain structures as a function of the number of neurons they contain in each species. **(C)** Variation in mass of cerebral cortex (circles), cerebellum (squares), and remaining brain structures (triangles) across species as a function of their numbers of non-neuronal (other) cells. Elephant data points shown in black (e), other afrotherian species in blue, primates in red (human, h), eulipotyphlans in orange, and glires in green. Data from (Herculano-Houzel et al., 2006, 2007, 2011; Azevedo et al., 2009; Sarko et al., 2009; Gabi et al., 2010; Neves et al., 2014).

Compared to other Afrotheria (Neves et al., 2014), the mammalian superorder to which elephants belong, the elephant cerebral cortex has the expected number of neurons for its mass (Figure 1A, circles). In contrast, for an afrotherian (Neves et al., 2014), the elephant cerebellum has a smaller mass than expected for its number of neurons (or more neurons than expected for its mass; Figure 1A, squares), and the rest of the brain has a larger mass than expected for its number of neurons (Figure 1B). All brain structures of the African elephant follow the same relationship between structure mass and numbers of other, non-neuronal, cells that applies to all mammalian species examined to date (Figure 1C). This indicates that the non-neuronal (presumably mostly glial) composition of the elephant brain conforms to the rules that are shared amongst all mammalian species analyzed to date, which appear to have been conserved in evolution (Herculano-Houzel, 2011a). In contrast, while the elephant cerebral cortex conforms to the neuronal scaling rules that apply to other afrotherians (Neves et al., 2014), its cerebellum has deviated markedly in evolution, as explored further below.

**Figure 2:**
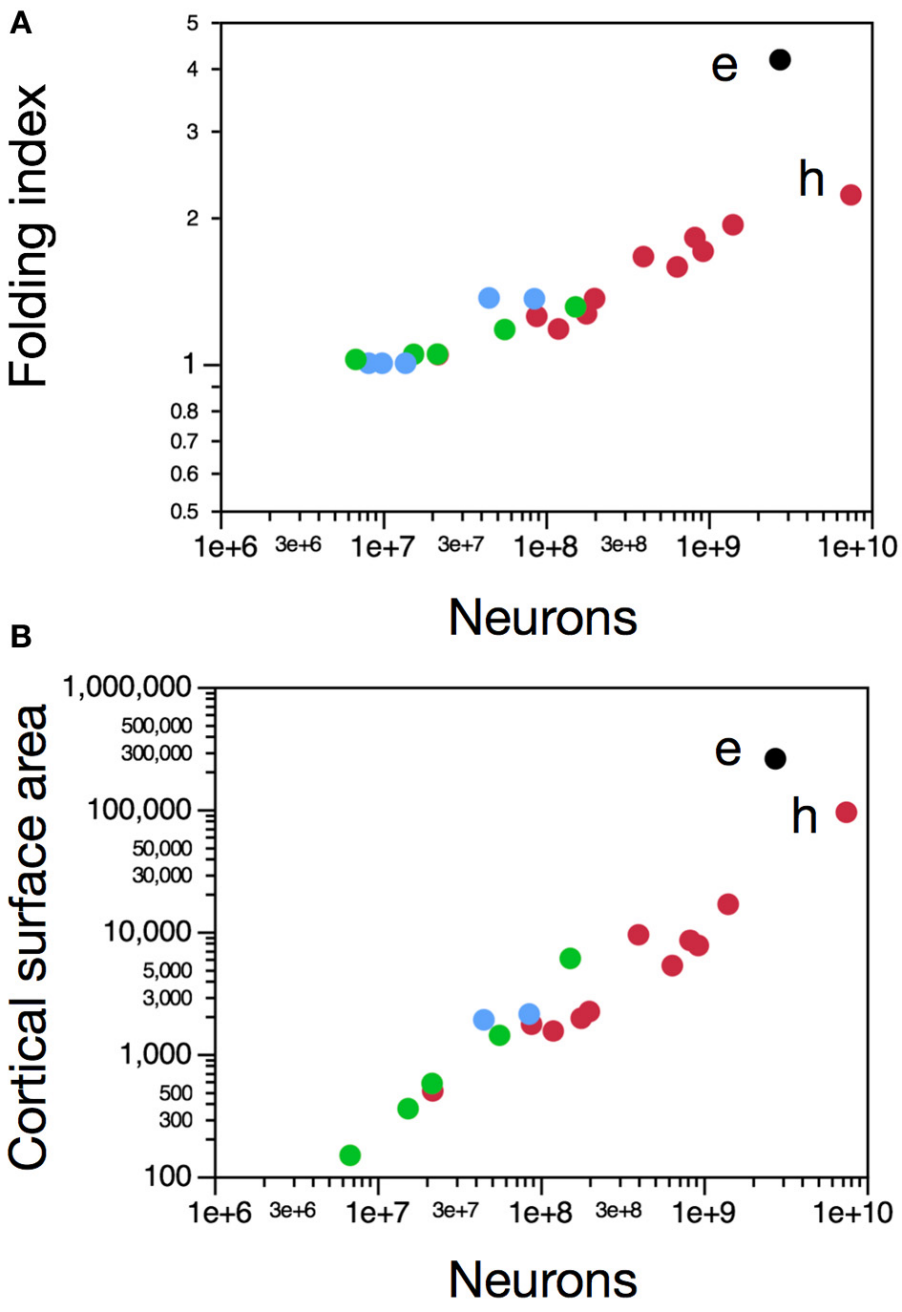
**Neurons in the elephant cerebral cortex are more spread out laterally, resulting in a more highly folded cortex than in other species with more cortical neurons**. Each data point represents values for one cerebral hemisphere in one species. Graphs show variation in folding index **(A)** and total surface area of the cerebral cortex **(B)** as a function of the number of neurons in the cerebral cortex in the elephant (black, e) and other species [other afrotherian species in blue, primates in red (human, h), and glires in green]. Data from Neves et al. (2014) and Ventura-Antunes et al. (2013).

With a folding index of 4.18, the elephant cerebral cortex is much more folded than both a hypothetical primate cortex with a similar number of neurons and the human cerebral cortex (Figure 2A), which corroborates our previous observation that the cortical folding index is not a simple function of the number of cortical neurons (Ventura-Antunes et al., 2013). Cortical neurons are spread laterally over 257,067 mm^2^ in the elephant, (Table 2) a much larger cortical surface than in primates with a similar number of cortical neurons (Figure 2B). As a consequence, the average number of neurons under 1 mm^2^ in the elephant cortex is only 10,752, compared to 80,064 in the human cerebral cortex (Ribeiro et al., 2013) or as much as 122,998 in the squirrel monkey (Herculano-Houzel et al., 2008). This large discrepancy across species demonstrates that, contrary to the notion put forward from extrapolations from two-dimensional counts limited to small portions of the cortex (Rockel et al., 1980) and later confirmed by stereology in a limited sample of 5 species (Carlo and Stevens, 2013), the number of neurons under a unit surface area of cerebral cortex is not constant across species. Our finding of largely disparate average numbers of neurons underneath 1 mm^2^ of cerebral cortex in the elephant compared to the human brain (Ribeiro et al., 2013), to other primates (Herculano-Houzel et al., 2008) and to rodents (Ventura-Antunes et al., 2013) adds to an already existing and growing body of evidence across insectivores (Stolzenburg et al., 1989), cetaceans (Poth et al., 2005) and a variety of species (Haug, 1987). Indeed, Haug (Haug, 1987) found about 15,000 neurons per mm^2^ in the cerebral cortex of the African elephant, a value fairly similar to that reported here. The non-uniformity of the number of neurons per surface area of the cerebral cortex has important implications for developmental models of cortical evolution in that it implies that cortical expansion can no longer be considered to occur through simple lateral addition of identical columnar modules (Rakic, 1988). Rather, neurons can spread underneath the cortical surface in different surface densities across species, which further implies that the expansion of the cortical surface, and also increasing degrees of cortical folding, are not synonym with expanding numbers of cortical neurons (Ventura-Antunes et al., 2013; Kazu et al., under review). Instead, we propose that cortical folding is a simple physical consequence of the expansion of a cortical surface under tension where axons are unequally distributed within the gray matter and through the white matter, regardless of the numbers of neurons that compose that surface (Mota and Herculano-Houzel, in preparation). In that scenario, we find that the degree of cortical folding of the African elephant, as that of all other mammals examined, is that expected for its surface area (Mota and Herculano-Houzel, in preparation).

**Table 2:** **Cortical areas and volumes in the African elephant**.

Total cortical surface area, pial	257,067 mm^2^
Exposed cortical surface area	62,146 mm^2^
Average folding index	4.18
Average cortical thickness	2.663 mm
Cortical surface area, interface with WM	208,633 mm^2^
Cortical volume, gray matter	676,937 mm^3^
Cortical volume, white matter	674,708 mm^3^

*Values for one hemisphere only*.

**Figure 3:**
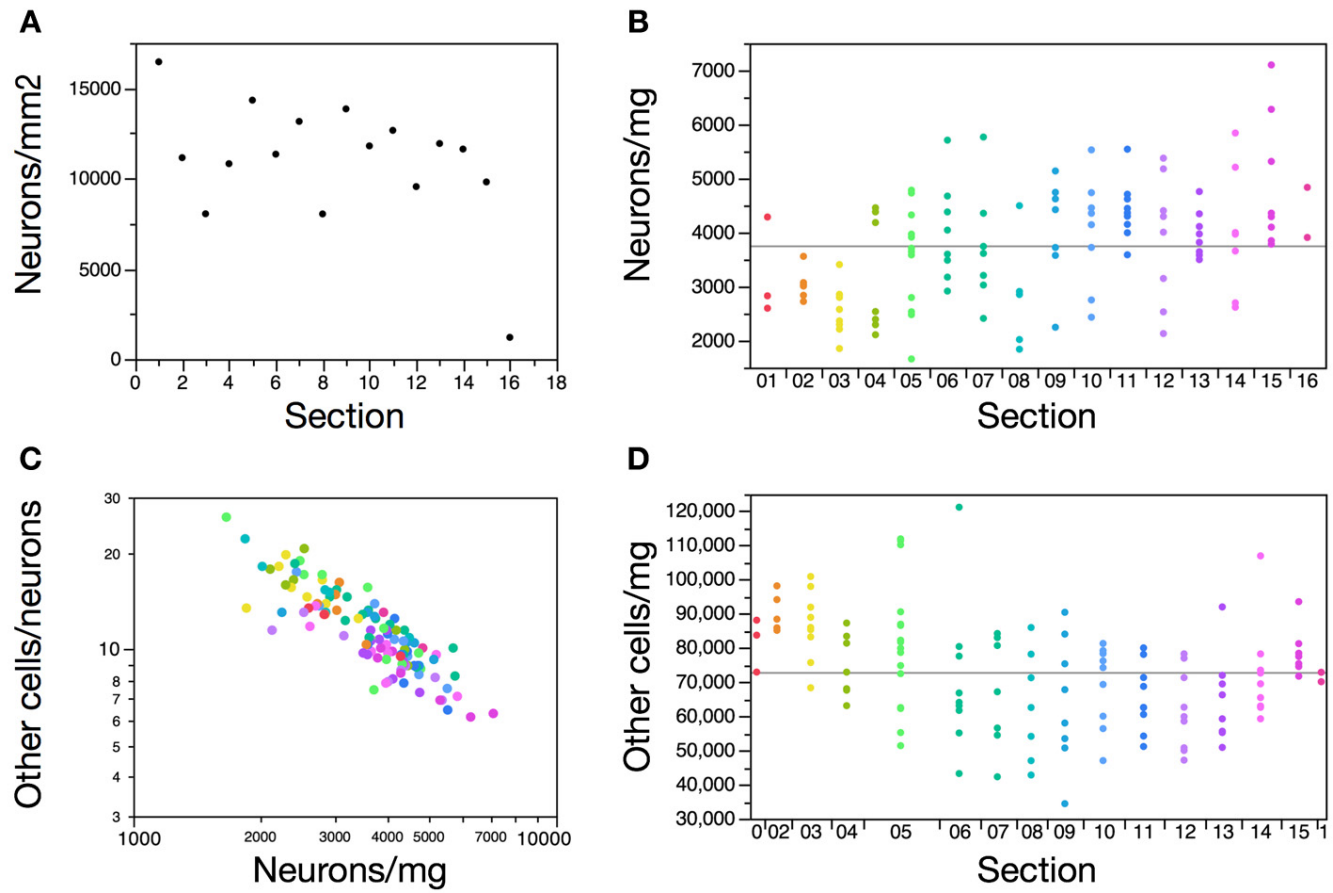
**Distribution of neurons along the anteroposterior axis of the elephant cerebral cortex**. **(A)** Average number of neurons under 1 mm^2^ of cerebral cortical surface varies randomly along the 16 coronal sections along the anteroposterior axis. Frontal pole is section 1. **(B)** Neuronal density in the cortical gray matter varies along the anteroposterior axis, with smaller neuronal densities in anterior sections 2–5 than in the remainder of the cortex. Each data point represents neuronal density in one piece of tissue within the section. **(C)** Ratio between numbers of other cells and neurons along the cortical gray matter decreases with increasing neuronal density. **(D)** Cell density in the subcortical white matter varies along the anteroposterior axis, with larger densities in anterior sections 1–5 than in the remainder of the white matter. Each data point represents cell density in one piece of tissue within the section.

Along the anteroposterior axis of the cerebral cortex, we find a 1.7-fold variation in the number of neurons under 1 mm^2^ of cortical surface (excluding the anterior and posterior poles), but with no correlation with position along the axis (Spearman correlation, *p* = 0.8170), contrary to what has been found in the human and non-human primate cortex (Cahalane et al., 2012; Ribeiro et al., 2013) (Figure 3A). Average neuronal density in the elephant cerebral cortical gray matter, at 3,661 neurons/mg, is small compared to other mammals, for example 21,450 in the human cortical gray matter and up to 122,232 neurons/mg in the mouse cortex (Ventura-Antunes et al., 2013), but it close to the ca. 5,000 neurons/mm^3^ reported previously for the African elephant (Haug, 1987). Given an average density of other cells of 38,910 per mg, which is comparable to that found in other mammalian cortices (Herculano-Houzel, 2011b) and to an average glial cell density of ca. 48,000 per mm^3^ reported previously (Haug, 1987), the low neuronal density in the elephant cerebral cortical gray matter indicates that neurons (including soma and all arborizations) are, on average, 10–40 times larger within the elephant cortical gray matter than in other mammalian cortices.

**Figure 4:**
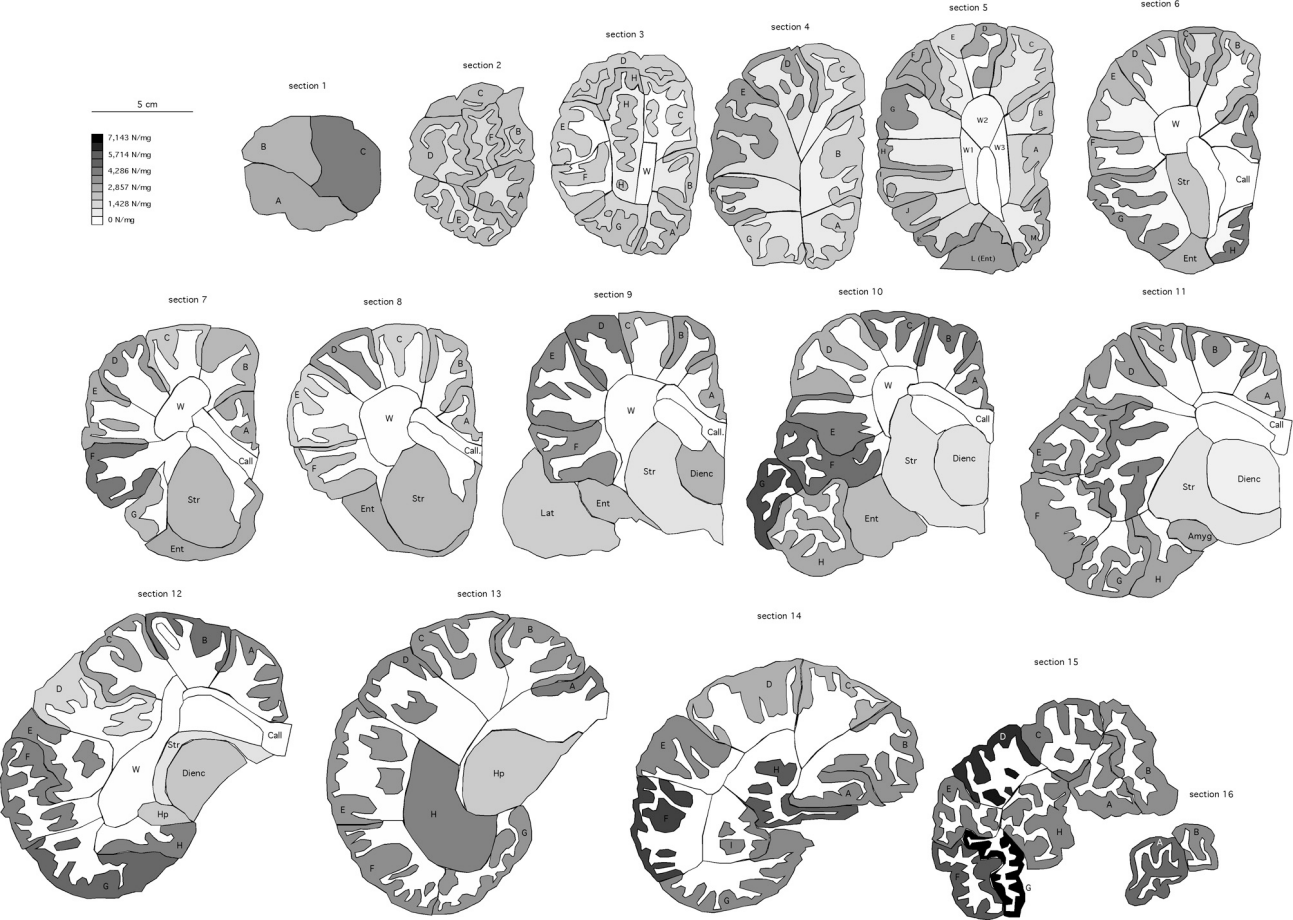
**Distribution of neuronal densities along the cerebral cortex of the African elephant**. Coronal sections, 1.28 mm thick, are numbered from the anterior to the posterior pole. Color intensity of the delineated cortical grey matter indicates the local neuronal density according to the scale on the left. A-I, blocks of cortical grey matter processed separately. W, white matter. Call, corpus callosum, processed separately from the remaining white matter. Amyg, amygdala; Dienc, diencephalon; Ent, entorhinal cortex; Hp, hippocampus; Str, striatum.

Neuronal densities (in neurons/mg) vary 4.3-fold along the 116 pieces of cerebral cortical gray matter [and include densities reported previously as the average neuronal density in the cerebral cortex of the African elephant (Haug, 1987) and in select locations of the cortex of the Indian elephant (Tower, 1954); Figure 4], and are smaller in sections 2–5, located in the anterior end of the series of 16 sections (Kruskal-Wallis test, *p* = 0.0019; Figure 3B). These findings are consistent with a larger average size of neurons (including soma, axons and dendrites) in the anterior-most portion of the elephant cerebral cortex (Jacobs et al., 2011), as has been seen in primates (Elston et al., 2001), and suggest that the distribution of neurons is not homogeneous along the cortical volume, although it does not vary as a single, continuous gradient along the anteroposterior axis.

The ratio between numbers of other cells and neurons varies as a function of neuronal density in the gray matter, as found within the human and mouse cerebral cortex (Herculano-Houzel et al., 2013; Ribeiro et al., 2013) (Figure 3C). This is consistent with our previous findings that indicate an evolutionarily conserved distribution of other cells as a function of neuronal density that is shared across brain structures and species (Herculano-Houzel, 2011b).

Other cell densities in the white matter vary 3.5-fold along the 128 pieces of white matter, and are larger in sections 1–5 and 15 along the 16 sections of cerebral cortex (Figure 3D). Because the vast majority of these cells are expected to be myelinating oligodendrocytes, this finding is consistent with a smaller caliber of myelinated fibers in the anterior-most than in the posterior-most sections of the elephant cortex, as found in the human cerebral cortex (Ribeiro et al., 2013).

**Figure 5:**
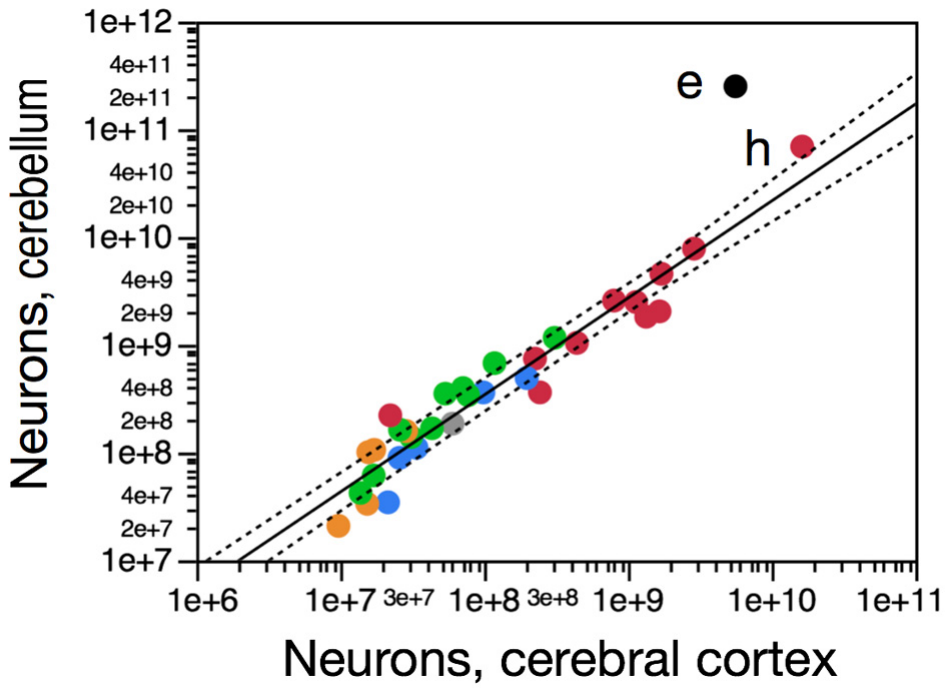
**The African elephant brain has a 10 times larger ratio between cerebellar and cortical neurons than other mammalian species**. Figure shows how number of neurons in the cerebellum varies homogeneously as a function of number of neurons in the cerebral cortex across species. Elephant data point shown in black (e), other afrotherian species in blue, primates in red (human, h), eulipotyphlans in orange, and glires in green. Data from (Herculano-Houzel et al., 2006, 2007, 2011; Azevedo et al., 2009; Sarko et al., 2009; Gabi et al., 2010; Neves et al., 2014).

The elephant cerebellum is relatively the largest found in mammals, comprising on average about 18% of brain volume (Maseko et al., 2012), while in other mammalian species the cerebellum usually represents between 10 and 14% of brain volume or mass (Clark et al., 2001; Herculano-Houzel, 2010; Maseko et al., 2012). In the elephant brain examined here, the cerebellum amounts to 25.4% of total brain mass. We had previously found the number of neurons in the cerebellum to vary as a common function of the number of neurons in the cerebral cortex across primates, rodents, and eulipotyphlans, and to hold on average 4.2 times more neurons than the cerebral cortex, reaching at most an average of 7.2 times more neurons in the cerebellum than in the cerebral cortex in eulipotyphlans (Herculano-Houzel, 2010). The African elephant is a clear outlier to this relationship, with a ratio of 44.8 neurons in the cerebellum to each neuron in the cerebral cortex (Figure 5).

The relatively large size of the African elephant cerebellum could be due to a relative decrease of the size of the cerebral cortex and/or remaining brain structures, or to an actual enlargement of the cerebellum. In view of our finding that the elephant cerebral cortex fits the neuronal scaling rules for Afrotherians (Neves et al., 2014) (Figure 1A), the elevated ratio between cerebellar and cortical neurons indicates that the elephant indeed has an increased number of neurons in the cerebellum, compared to the cerebral cortex. Moreover, the discrepancy between the number of neurons in the elephant cerebellum and that expected for an afrotherian cerebellum of its size (Figure 1A) indicates that the increase in numbers of cerebellar neurons in the African elephant did not obey the scaling rules that apply to other afrotherians; that is, the elephant cerebellum is not simply a scaled-up afrotherian cerebellum. Rather, the finding that the elephant cerebellum is smaller than expected for an afrotherian, for its number of neurons, suggests a selective pressure for compactedness. Thus, the elephant cerebellum seems to be twice specialized: it has both an increased number of neurons relative to the cerebral cortex, and a more compact packing of neurons than found in other afrotherians. Although it cannot be determined at this point whether the extraordinarily large number of neurons in the cerebellum is due to addition of all cell types or particularly to granule cells, a previous analysis of the morphology of the African elephant cerebellum showed no departure from the typical cerebellar organization of other mammals (Maseko et al., 2012). However, given that granule cells are the vast majority of neurons in the mammalian cerebellum, including in the elephant (Maseko et al., 2012), the increased number of neurons in the elephant cerebellum must include the addition of vast numbers of granule cell neurons, even if not exclusively of this cell type.

The African elephant thus has a ca. 10-fold larger ratio of cerebellar to cerebral cortical neurons compared to all other mammals analyzed previously (Herculano-Houzel, 2010). The concerted scaling of numbers of neurons in the cerebellar and cerebral cortices, maintaining an average ratio of 4.2 between the structures, supports the view of coordinated function of the two cortices, which are functionally coupled (Leiner et al., 1989; Ramnani, 2006; Ito, 2008) and scale concertedly in absolute mass across species of several mammalian orders (Stephan et al., 1981; Whiting and Barton, 2003). Cerebellar function is now known not to be limited to somatomotor processing, and to involve loops with the entire cerebral cortex, with remarkably extensive processing of prefrontal cortical information that contribute to cognitive functions as well (Buckner, 2013). Indeed, in primate evolution there has been a concerted increase in the volume of the prefrontal cortex, of prefrontal inputs to the cortico-pontine system, and of prefrontal-projecting cerebellar lobules (Ramnani et al., 2006; Balsters et al., 2010).

If such functional coupling also exists in the African elephant, it is at odds with the much larger number of neurons in the cerebellum than expected for the number of neurons in the cerebral cortex. Although studies of anatomical connectivity between the cerebellum and cerebral cortex in the elephant will be necessary to address this possibility, this discrepancy suggests that the massive addition of supernumerary neurons to the cerebellum is not related to cerebral cortical processing, but rather to processing of afferent information that arrives from non-cortical sources, such as the brainstem. Two candidate and non-exclusive sources of unusual amounts of afferent information to be processed by the elephant cerebellum are those related to infrasound communication and to the trunk.

Elephants, odontocete cetaceans, and microchiropterans have relatively larger cerebella than other mammals (Maseko et al., 2012), and all three groups share unusual systems of vocalization: infrasound in elephants (Garstang, 2004), and echolocation in cetaceans and microchiropterans (Rendell et al., 1999; Speakman, 2001). The exact mechanism for the production of infrasound in elephants is currently unknown, but is presumed to involve the larynx and trunk (Herbst et al., 2012; Stoeger et al., 2012). It is thus possible that the enlarged cerebellum is related to vocalization; however, we have found that, in contrast to the elephant, the relatively large cerebellum of echolocating microchiropterans is not an enlarged cerebellum, but rather the result of a diminished cerebral cortex, possibly related to miniaturization (Herculano-Houzel et al., in preparation). This finding suggests that the neuron-rich, complex cerebellar neuronal morphology (Maseko et al., 2013a) and enlarged cerebellum (Maseko et al., 2012) of the African elephant is not related to vocalization.

Alternatively, the increased number of cerebellar neurons in the elephant might be related to the trunk, a highly tactile and motile muscular appendage (Rasmussen and Munger, 1996) that in principle has infinite degrees of freedom of movement, given the absence of internal bones and joints. The highly sensitive tip of the trunk moves extensively as the elephant explores and manipulates its immediate environment. We speculate that the extraordinary number of neurons in the elephant cerebellum, and thus the large absolute and relative size of this structure (Maseko et al., 2012) as well as brainstem motor specializations (Maseko et al., 2013b), may have been driven by the processing of complex sensory and motor information regarding the trunk, a large sensorimotor unpaired appendage unique to the Proboscideans.

In contrast to its exceptional cerebellum, here we show that the cerebral cortex of the African elephant, with 5.6 billion neurons [a value that is similar to the ca. 7–8 billion neurons estimated from the multiplication of neuronal density and cortical volume (Haug, 1987)], matches the neuronal composition expected for an afrotherian (Neves et al., 2014), which differs from that expected for a primate of similar cortical mass (Herculano-Houzel, 2011a). Thus, as predicted previously (Herculano-Houzel, 2009), the elephant cerebral cortex has far fewer neurons than the human cerebral cortex (Azevedo et al., 2009), and fewer than estimated for the great ape cerebral cortex (Herculano-Houzel and Kaas, 2011) (ca. 9 billion neurons in gorilla and orangutan, 8 billion in the chimpanzee cerebral cortex), although more than in macaque monkeys and in the baboon (1–3 billion neurons Gabi et al., 2010). It is tempting to speculate that the cognitive abilities shared by great apes and elephants, such as cooperation and mirror-recognition (Plotnik et al., 2006, 2011; Hart et al., 2008), are related to their numbers of cortical neurons, which are fewer than in the human cortex but still more than found in many other mammals, including macaques and large artiodactyls (Kazu et al., under review). It remains to be seen how numbers of neurons in prefrontal (associative) cortical areas compare across these species, as well as numbers of synapses and patterns of connectivity. In the meantime, the present data lend weight to the hypothesis that the simplest explanation, based on data gathered to date, for the remarkable cognitive abilities of the human brain lies in the remarkable number of neurons concentrated in the cerebral cortex, which is the expected number for a primate brain of human proportions (Herculano-Houzel, 2012).

## Methods

We analyzed the right half of the brain of one adult male specimen of *Loxodonta africana* obtained as part of a planned and authorized cull permitted by the Zimbabwe Parks and Wildlife Management Authority, with ethical permission from the University of the Witwatersrand Animal Ethics Committee (Manger et al., 2009). Additionally, permission was granted by the Malilangwe Nature Conservation Trust to obtain this animal on their property near Chiredzi in southeastern Zimbabwe. All work was performed under the direction and supervision of an experienced wildlife veterinarian employed by the Malilangwe Trust.

The half-brain analyzed belonged to elephant LA2, with a total post-perfusion brain mass (including pia and arachnoid mater and fluid) of 5250 g (Manger et al., 2009). The animal was perfused onsite, and the brain was post-fixed in a 4% solution of phosphate-buffered paraformaldehyde for 72 h, then stored in antifreeze solution at −20°C until processed.

The right half of the brain was parceled into 381 different pieces of tissue, which were processed separately. We first separated the olfactory bulb by transecting the olfactory tract immediately proximal to the bulb, and separated the cerebellum by cutting the cerebellar peduncles at the surface of the brainstem, then sectioned it manually into 8 sagittal sections of 1.28 mm thickness each. The brainstem was isolated by cutting through a plane anterior to the colliculi and posterior to the hypothalamus, and separated into mesencephalon, pons, and medulla, which were further subdivided for processing. The remaining cerebrum was then sectioned manually into 16 coronal sections of 1.28 mm each. Each section was scanned at 300 dpi for reconstruction of the surface. From each section, we then dissected the corpus striatum, diencephalon, hippocampus, amygdala, and entorhinal cortex. The remaining cerebral cortex was further dissected into smaller parts and separated into gray and white matter for cell counting using the isotropic fractionator (Herculano-Houzel and Lent, 2005).

Cortical surface areas were estimated for each section by tracing the cortical gray and white matter surfaces in NeuroLucida (Microbrightfield Bioscience, VT). Cortical gray and white matter coronal surface areas were estimated in the same program by applying Cavalieri analysis with a grid size of 1 mm. Surface area and volume for the entire cortex were then estimated by using a formula that corrects for cortical curvature, as described in Ribeiro et al. (2013). The folding index of the cerebral cortex was calculated as the ratio between the total pial surface of the gray matter and the exposed surface of the cortex. Data analysis was performed with JMP 9.0 software (SAS, USA). All numbers reported correspond to the numbers obtained for the right half-brain multiplied by 2 for comparison with published data for the whole brain for other species.

## Author contributions

Paul R. Manger collected tissue, Suzana Herculano-Houzel and Paul R. Manger designed research, Suzana Herculano-Houzel, Kamilla Avelino-de-Souza, Kleber Neves, Jairo Porfírio, Larissa Mattos Feijó and Débora Messeder processed tissue, Suzana Herculano-Houzel and José Maldonado analyzed data, Suzana Herculano-Houzel and Paul R. Manger wrote the paper.

### Conflict of interest statement

José Maldonado works for Microbrightfield, who commercializes one of the softwares employed in the study. The authors declare that the research was conducted in the absence of any commercial or financial relationships that could be construed as a potential conflict of interest.

## References

[B1] Andrade-Moraes C. H. Oliveira-Pinto A. V. Castro-Fonseca E. da Silva C. G. Guimarães D. M. Szczupak D. (2013). Cell number changes in Alzheimer's disease relate to dementia, not to plaques and tangles. Brain 136, 3738–3752 10.1093/brain/awt273 24136825 PMC3859218

[B2] Azevedo F. A. Carvalho L. R. Grinberg L. T. Farfel J. M. Ferretti R. E. Leite R. E. (2009). Equal numbers of neuronal and nonneuronal cells make the human brain an isometrically scaled-up primate brain. J. Comp. Neurol. 513, 532–541 10.1002/cne.21974 19226510

[B3] Bahney J. von Bartheld C. S. (2014). Calibration of the isotropic fractionator: comparison with unbiased stereology and DNA extraction for quantification of glial cells. J. Neurosci. Methods 222, 165–174 10.1016/j.jneumeth.2013.11.002 24239779 PMC3904371

[B4] Balsters J. H. Cussans E. Diedrichsen J. Phillips K. A. Preuss T. M. Rilling J. K. (2010). Evolution of the cerebellar cortex: the selective expansion of prefrontal-projecting cerebellar lobules. Neuroimage 49, 2045–2052 10.1016/j.neuroimage.2009.10.045 19857577 PMC6436533

[B5] Buckner R. L. (2013). The cerebellum and cognitive function: 25 yers of insight from anatomy and neuroimaging. Neuron 80, 807–815 10.1016/j.neuron.2013.10.044 24183029

[B6] Cahalane D. J. Charvet C. J. Finlay B. F. (2012). Systematic, balancing gradients in neuron density and number across the primate isocortex. Front. Neuroanat. 6:28 10.3389/fnana.2012.00028 22826696 PMC3399120

[B7] Carlo C. N. Stevens C. S. (2013). Structural uniformity of neocortex, revisited. Proc. Natl. Acad. Sci. U.S.A. 110, 1488–1493 10.1073/pnas.1221398110 23297199 PMC3557031

[B8] Clark D. A. Mitra P. P. Wang S. S. (2001). Scalable architecture in mammalian brains. Nature 411, 189–193 10.1038/35075564 11346794

[B9] Deaner R. O. Isler K. Burkart J. van Schaik C. (2007). Overall brain size, and not encephalization quotient, best predicts cognitive ability across non-human primates. Brain Behav. Evol. 70, 115–124 10.1159/000102973 17510549

[B10] Elston G. N. Benavides-Piccione R. DeFelipe J. (2001). The pyramidal cell in cognition: a comparative study in human and monkey. J. Neurosci. 21, RC163 11511694 10.1523/JNEUROSCI.21-17-j0002.2001 PMC6763111

[B11] Gabi M. Collins C. E. Wong P. Torres L. B. Kaas J. H. Herculano-Houzel S. (2010). Cellular scaling rules for the brains of an extended number of primate species. Brain Behav. Evol. 76, 32–44 10.1159/000319872 20926854 PMC2980814

[B12] Garstang M. (2004). Long-distance, low-frequency elephant communication. J. Comp. Physiol. A Neuroethol. Sens. Neural. Behav. Physiol. 190, 791–805 10.1007/s00359-004-0553-0 15349746

[B13] Hart B. L. Hart L. A. Pinter-Wollman N. (2008). Large brains and cognition: where do elephants fit in? Neurosci. Biobehav. Rev. 32, 86–98 10.1016/j.neubiorev.2007.05.012 17617460

[B14] Haug H. (1987). Brain sizes, surfaces, and neuronal sizes of the cortex cerebri: a stereological investigation of man and his variability and a comparison with some mammals (primates, whales, marsupials, insectivores, and one elephant). Am. J. Anat. 180, 126–142 10.1002/aja.1001800203 3673918

[B15] Herbst C. T. Stoeger A. S. Frey R. Lohscheller J. Titze I. R. Gumpenberger M. (2012). How low can you go? Physical production mechanism of elephant infrasonic vocalizations. Science 337, 595–599 10.1126/science.1219712 22859490

[B16] Herculano-Houzel S. (2009). The human brain in numbers: a linearly scaled-up primate brain. Front. Hum. Neurosci. 3:31 10.3389/neuro.09.031.2009 19915731 PMC2776484

[B17] Herculano-Houzel S. (2010). Coordinated scaling of cortical and cerebellar numbers of neurons. Front. Neuroanat. 4:12 10.3389/fnana.2010.00012 20300467 PMC2839851

[B18] Herculano-Houzel S. (2011a). Brains matter, bodies maybe not: the case for examining neuron numbers irrespective of body size. Ann. Rev. N.Y. Acad. Sci. 1225, 191–199 10.1111/j.1749-6632.2011.05976.x 21535005

[B19] Herculano-Houzel S. (2011b). Not all brains are made the same: new views on brain scaling in evolution. Brain Behav. Evol. 78, 22–36 10.1159/000327318 21691045

[B20] Herculano-Houzel S. (2012). The remarkable, yet not extraordinary, human brain as a scaled-up primate brain and its associated cost. Proc. Natl. Acad. Sci. U.S.A. 109, 10661–10668 10.1073/pnas.1201895109 22723358 PMC3386878

[B16b] Herculano-Houzel S. Collins C. Wong P. Kaas J. H. (2007). Cellular scaling rules for primate brains. Proc. Natl. Acad. Sci. U.S.A. 104, 3562–3567 10.1073/pnas.0611396104 17360682 PMC1805542

[B21] Herculano-Houzel S. Collins C. Wong P. Kaas J. H. Lent R. (2008). The basic nonuniformity of the cerebral cortex. Proc. Natl. Acad. Sci. U.S.A. 105, 12593–12598 10.1073/pnas.0805417105 18689685 PMC2527956

[B22] Herculano-Houzel S. Kaas J. H. (2011). Gorilla and orangutan brains conform to the primate scaling rules: implications for hominin evolution. Brain Behav. Evol. 77, 33–44 10.1159/000322729 21228547 PMC3064932

[B23] Herculano-Houzel S. Lent R. (2005). Isotropic fractionator: a simple, rapid method for the quantification of total cell and neuron numbers in the brain. J. Neurosci. 25: 2518–2521 10.1523/JNEUROSCI.4526-04.2005 15758160 PMC6725175

[B16a] Herculano-Houzel S. Mota B. Lent R. (2006). Cellular scaling rules for rodent brains. Proc. Natl. Acad. Sci. U.S.A. 103, 12138–12143 10.1073/pnas.0604911103 16880386 PMC1567708

[B16c] Herculano-Houzel S. Ribeiro P. F. M. Campos L. da Silva A. V. Torres L. B. Catania K. C. (2011). Updated neuronal scaling rules for the brains of Glires (rodents/lagomorphs). Brain Behav. Evol. 78, 302–314 10.1159/000330825 21985803 PMC3237106

[B24] Herculano-Houzel S. Watson C. Paxinos G. (2013). Distribution of neurons in functional areas of the mouse cerebral cortex reveals quantitatively different cortical zones. Front. Neuroanat. 7:35 10.3389/fnana.2013.00035 24155697 PMC3800983

[B25] Ito M. (2008). Control of mental activities by internal models in the cerebellum. Nat. Rev. Neurosci. 9, 304–313 10.1038/nrn2332 18319727

[B26] Jacobs B. Lubs J. Hannan M. Anderson K. Butti C. Sherwood C. (2011). Neuronal morphology in the African elephant (*Loxodonta africana*) neocortex. Brain Struct. Funct. 215, 273–298 10.1007/s00429-010-0288-3 21079992

[B27] Jerison H. J. (1973). Evolution of the Brain and Intelligence. New York, NY: Academic Press

[B28] Leiner H. C. Leiner A. L. Dow R. S. (1989). Reappraising the cerebellum: what does the hindbrain contribute to the forebrain? Behav. Neurosci. 103, 998–1008 10.1037/0735-7044.103.5.998 2679667

[B29] Manger P. R. Pillay P. Maseko B. C. Bhagwandin A. Gravett N. Moon D. (2009). Acquisition of the brain of the African elephant (Loxodonta africana): perfusion-fixation and dissection. J. Neurosci. Methods 179, 16–21 10.1016/j.jneumeth.2009.01.001 19168095

[B30] Marino L. (1998). A comparison of encephalization between odontocete cetaceans and anthropoid humans. Brain Behav. Evol. 51, 230–238 10.1159/000006540 9553695

[B31] Maseko B. Jacobs B. Spocter M. Sherwood C. Hof P. Manger P. (2013a). Qualitative and quantitative aspects of the microanatomy of the African elephant cerebellar cortex. Brain Behav. Evol. 81, 40–55 10.1159/000345565 23296570

[B32] Maseko B. Patzke N. Fuxe K. Manger P. (2013b). Architectural organization of the African elephant diencephalon and brainstem. Brain Behav. Evol. 82, 83–128 10.1159/000352004 24021932

[B33] Maseko B. C. Spocter M. A. Haagensen M. Manger P. R. (2012). Elephants have relatively the largest cerebellum size of mammals. Anat. Rec. 295, 661–672 10.1002/ar.22425 22282440

[B34] Neves K. Jr. Meireles F. F. Tovar-Moll F. Gravett N. Bennett N. C. Kaswera C. (2014). Cellular scaling rules for the brain of afrotherians. Front. Neuroanat. 8:5 10.3389/fnana.2014.00005 24596544 PMC3925844

[B35] Patzke N. Olaleye O. Haagensen M. Hof P. R. Ihunwo A. O. Manger P. R. (2013). Organization and chemical neuroanatomy of the African elephant (*Loxodonta africanus*) hippocampus. Brain Struct. Funct. 216, 403–416 10.1007/s00429-013-0587-6 23728481

[B36] Plotnik J. M. de Waal F. B. M. Reiss D. (2006). Self-recognition in an Asian elephant. Proc. Natl. Acad. Sci. U.S.A. 103, 17053–17057 10.1073/pnas.0608062103 17075063 PMC1636577

[B37] Plotnik J. M. Lair R. Suphachoksahakun W. de Waal F. B. M. (2011). Elephants know when they need a helping trunk in a cooperative task. Proc. Natl. Acad. Sci. U.S.A. 108, 5116–5121 10.1073/pnas.1101765108 21383191 PMC3064331

[B38] Poth C. Fung C. Güntürkün S. H. Ridgwat S. H. Oelschläger H. H. A. (2005). Neuron numbers in sensory cortices of five delphinids compared to a physeterid, the pygmy sperm whale. Brain Res. Bull. 66, 357–360 10.1016/j.brainresbull.2005.02.001 16144614

[B39] Premack D. (2007). Human and animal cognition: continuity and discontinuity. Proc. Natl. Acad. Sci. U.S.A. 104, 13861–13867 10.1073/pnas.0706147104 17717081 PMC1955772

[B40] Rakic P. (1988). Specification of cerebral cortical areas. Science 241, 170–176 10.1126/science.3291116 3291116

[B41] Ramnani N. (2006). The primate cortico-cerebellar system: anatomy and function. Nat. Rev. Neurosci. 7, 511–522 10.1038/nrn1953 16791141

[B42] Ramnani N. Behrens T. E. Johansen-Berg H. Richter M. C. Pinsk M. A. Andersson J. L. (2006). The evolution of prefrontal inputs to the cortico-pontine system: diffusion imaging evidence from macaque monkeys and humans. Cereb. Cortex 16, 811–818 10.1093/cercor/bhj024 16120793

[B43] Rasmussen L. E. Munger B. L. (1996). The sensorial specializations of the trunk tip (finger) of the Asian elephant, *Elephas maximus*. Anat. Rec. 246, 127–134 8876831 10.1002/(SICI)1097-0185(199609)246:1<127::AID-AR14>3.0.CO;2-R

[B44] Rendell L. E. Matthews J. N. Gill A. Gordon J. C. D. Macdonald D. W. (1999). Quantitative analysis of tonal calls from five odontocete species, examining interspecific and intraspecific variation. J. Zool. 249, 403–410 10.1111/j.1469-7998.1999.tb01209.x

[B45] Ribeiro P. F. M. Ventura-Antunes L. Gabi M. Mota B. Grinberg L. T. Farfel J. M. (2013). The human cerebral cortex is neither one nor many: neuronal distribution reveals two quantitatively different zones in the grey matter, three in the white matter, and explains local variations in cortical folding. Front. Neuroanat. 7:28 10.3389/fnana.2013.00028 PMC3759024 24032005

[B46] Rockel A. J. Hiorns R. W. Powell T. P. (1980). The basic uniformity in structure of the neocortex. Brain 103, 221–244 10.1093/brain/103.2.221 6772266

[B47] Roth G. Dicke U. (2005). Evolution of the brain and intelligence. Trends Cogn. Sci. 9, 250–257 10.1016/j.tics.2005.03.005 15866152

[B47a] Sarko D. K. Catania K. C. Leitch D. B. Kaas J. H. Herculano-Houzel S. (2009). Cellular scaling rules of insectivore brains. Front. Neuroanat. 3:8 10.3389/neuro.05.008.2009 19636383 PMC2713736

[B48] Speakman J. R. (2001). The evolution of flight and echolocation in bats: another leap in the dark. Mammal. Rev. 31, 111–130 10.1046/j.1365-2907.2001.00082.x

[B49] Stephan H. Frahm H. Baron G. (1981). New and revised data on volumes of brain structures in insectivores and primates. Folia Primatol. 35, 1–29 10.1159/000155963 7014398

[B50] Stoeger A. S. Heilmann G. Zeppelzauer M. Ganswindt A. Hensman S. Charlton B. D. (2012). Visualizing sound emission of elephant vocalizations: evidence for two rumble production types. PLoS ONE 7:e48907 10.1371/journal.pone.0048907 23155427 PMC3498347

[B51] Stolzenburg J. U. Reichenbach A. Neumann M. (1989). Size and density of glial and neuronal cells within the cerebral neocortex of various insectivorian species. Glia 2, 78–84 10.1002/glia.440020203 2524445

[B52] Tower D. (1954). Structural and functional organization of mammalian cerebral cortex: the correlation of neurone density with brain size. Cortical neurone density in the fin whale (*Balaenoptera physalus* L.) with a note on the cortical neurone density in the Indian elephant. J. Comp. Neurol. 101, 19–51 10.1002/cne.901010103 13211853

[B53] Ventura-Antunes L. Mota B. Herculano-Houzel S. (2013). Different scaling of white matter volume, cortical connectivity, and gyrification across rodent and primate brains. Front. Neuroanat. 7:3 10.3389/fnana.2013.00003 23576961 PMC3620553

[B54] Whiting B. A. Barton R. A. (2003). The evolution of the cortico-cerebellar complex in primates: anatomical connections predict patterns of correlated evolution. J. Hum. Evol. 44, 3–10 10.1016/S0047-2484(02)00162-8 12604300

